# One-stage design is empirically more powerful than two-stage design for family-based genome-wide association studies

**DOI:** 10.1186/1753-6561-1-s1-s137

**Published:** 2007-12-18

**Authors:** Rori V Rohlfs, Chelsea Taylor, Lucia Mirea, Shelley B Bull, Mary Corey, Amy D Anderson

**Affiliations:** 1 Department of Genome Sciences, University of Washington, 1705 Northeast Pacific Street #K-357, Box 357730, Seattle, Washington 98195, USA; 2 Hospital for Sick Children Research Institute, 555 University Avenue, Toronto, Ontario M5G 1X8, Canada; 3 Department of Public Health Sciences, University of Toronto, 155 College Street, Toronto, Ontario, M5T 3M7, Canada; 4 Samuel Lunenfeld Research Institute, Mount Sinai Hospital, 60 Murray Street, Suite L5-227, Toronto, Ontario M5T 3L9, Canada; 5 Department of Biostatistics, University of Washington, Campus Box 357232, Seattle, Washington 98195, USA

## Abstract

Finding a genetic marker associated with a trait is a classic problem in human genetics. Recently, two-stage approaches have gained popularity in marker-trait association studies, in part because researchers hope to reduce the multiple testing problem by testing fewer markers in the final stage. We compared one two-stage family-based approach to an analogous single-stage method, calculating the empirical type I error rates and power for both methods using fully simulated data sets modeled on nuclear families with rheumatoid arthritis, and data sets of real single-nucleotide polymorphism genotypes from Centre d'Etude du Polymorphisme Humain pedigrees with simulated traits. In these analyses performed in the absence of population stratification, the single-stage method was consistently more powerful than the two-stage method for a given type I error rate. To explore the sources of this difference, we performed a case study comparing the individual steps of two-stage designs, the two-stage design itself, and the analogous one-stage design.

## Background

Linkage methods test for co-inheritance of a genetic marker and trait through pedigrees, while association studies test for correlations between a marker and trait at the population level. Fulker et al. [1] decomposed pedigree data into two independent sources of information: association and correlative transmission. Under this decomposition, expected children's genotypes given parental genotypes can be used in an association test, while actual children's genotypes can be used independently in a transmission test [2-5], leading naturally to a two-stage design.

It has recently been shown that for every two-stage multiple testing procedure there is a corresponding single-stage procedure that will identify a greater number of expected true positives for a fixed level of expected false positives [6]. Leek and Storey have also shown in unpublished work that a popular two-stage method for family-based association studies [2,4,5] can be improved by using an analogous single stage procedure. Here we provide an empirical comparison of 1) Lange's two-stage method that uses a conditional means model test (CMMT) in Stage 1 followed by the FBAT in Stage 2 (Lange et al. [4]), 2) a single stage method, the CSST, which combines the test statistics from the two stages of Lange's method, and 3) a single stage procedure (described in personal communications with Leek and Storey) on the Genetic Analysis Workshop 15 (GAW15) data. All three methods are described in the statistical tests section below.

## Methods

### Problem 3 data set

The GAW15 Problem 3 data consisted of 100 simulated data sets of affected families with genotype and quantitative phenotype information patterned after data from rheumatoid arthritis studies [7]. Due to data corruption issues and time constraints, we analyzed simulations 1–64, 75, 77–87, and 90–95. Each simulated data set consisted of 1500 two-child nuclear families. Every individual had genotype information for 9187 autosomal single-nucleotide polymorphisms (SNPs) and several phenotype measures. Having looked at the simulation "answers," we used latent severity as the trait of interest. Values of latent severity were simulated based on independent effects of two diallelic loci, not in linkage disequilibrium (LD), on chromosome 9. A quarter of the variability in latent severity was due to each locus independently and the rest was due to individual random effects.

### Problem 1 data set

The GAW15 Problem 1 data set consisted of a set of 14 CEPH (Centre d'Etude du Polymorphisme Humain) pedigrees in which individuals have been genotyped for 2819 autosomal SNPs [8]. For our analysis, we ignored grandparental data and looked only at nuclear families. We adopted a convention that a family's data was ignored for a SNP when a parental genotype was missing for that SNP. This allowed us to test transmission without confronting the issue of missing parental genotypes. The methods used here rely on asymptotic theory, so SNPs missing in more than 10% of the individuals or with a minor allele frequency less than 0.1 were removed from the data set, leaving 1509 autosomal SNPs.

In order to examine the behavior of the methods under various trait models on realistic data with known causal SNPs, we simulated traits based on the Problem 1 genotypes. We used two models for our simulations: a simple additive model and an additive model with polygenic effects (hereafter referred to the polygenic model). Simple additive traits follow the model *y*_*ij *_= *μ *+ *ax*_*ij *_+ *ε*_*ij*_, where *y*_*ij *_is the trait value of individual *j *from family *i*, *μ *is the average trait value, *a *is the genetic effect, *x*_*ij *_is the number of copies of allele *A *at the causal SNP in individual *j *from family *i*, and *ε*_*ij *_is a *N(0*, $\sigma_{e}^{2}$*) *residual. For polygenic traits, we used the model *y*_*ij *_= *μ *+ *ax*_*ij *_+ *z*_*ij *_+ *ε*_*ij*_, where in the parents, *z*_*ij *_are normal random variables with mean 0 and variance $\sigma_{p}^{2}$. Given parental polygenic effects *z*_*im *_and *z*_*if*_, the offspring polygenic values follow a *N*((*z*_*im *_+ *z*_*if*_)/2, $\sigma_{p}^{2}$/2) distribution.

### Statistical tests

For a quantitative trait, the conditional means model test (CMMT) described by Lange et al. [4] examines each SNP for association using the linear mixed-effect model:

*y*_*ij *_= *μ *+ *β*_0_*E*(*x*_*ij *_| *x*_*im*_, *x*_*if*_) + *z*_*i *_+ *ε*_*ij*_,

where *β*_0 _is the genetic effect, *E*(*x*_*ij *_| *x*_*im*_, *x*_*if*_) is the child's expected genotype given the parental genotypes, *z*_*i *_is a random family effect, and *ε *is residual error. The CMMT is the Wald statistic to test the null hypothesis of no association that specifies *β*_0 _= 0 vs. the alternative *β*_0 _≠ 0 [5]. Generalized estimating equations are used to fit the model and obtain *p*-values for each SNP.

The family-based association test (FBAT) [4] is a score statistic for transmission disequilibrium based on the model:

*y*_*ij *_= *μ*' + *β*'_0_(*x*_*ij *_- *E*(*x*_*ij *_| *x*_*im*_, *x*_*if*_)) + *ε*'_*ij*_,

where the null hypothesis is *β*'_0 _= 0 and the alternative is *β*'_0 _≠ 0. This test statistic follows a $\chi_{1}^{2}$ distribution in large samples.

The CMMT and FBAT provide statistically independent tests corresponding to between- and within-family association analyses of a SNP and quantitative trait [2,4,5]. These test statistics can be examined separately in two stages [4,5] or combined in a single-stage analysis. The two-stage method screens every SNP with the CMMT and passes the ten best-scoring SNPs to the second stage. Retaining the top ten SNPs is recommended by Van Steen et al. [5] based on estimates of power to identify multiple loci of small effects in simulations of a 10 K genome scan. In the second stage, an FBAT score statistic is computed for each of the ten retained SNPs and a Bonferroni correction factor of ten is applied. The corrected FBAT *p*-values are considered to be the final genome-wide *p*-values for those ten SNPs.

A single-stage analysis can be performed by adding the CMMT and FBAT tests statistics to obtain a chi-square sum test (CSST). Because both CMMT and FBAT are independent test statistics with an asymptotic $\chi_{1}^{2}$ distribution [5], the resulting CSST statistic has an asymptotic $\chi_{2}^{2}$ distribution.

Leek, Rohlfs, and Storey developed the conditional likelihood ratio test (CLRT) (personal communication), another single-stage method analogous to the two-stage method. As in Lange's two-stage method, the CLRT one-stage likelihood can be divided into association and linkage portions [2]. Note that:

*L *= *L*(*y*_*ij*_, *x*_*ij *_| *θ*, *θ*', *x*_*im*_, *x*_*if*_) = *L*(*y*_*ij *_| *θ*, *x*_*im*_, *x*_*if*_)*L*(*x*_*ij *_| *y*_*ij*_, *θ*', *x*_*im*_, *x*_*if*_),

where *θ *and *θ*' are vectors of model parameters. The screening step from the two-stage design is mirrored in *L*(*y*_*ij *_| *θ*, *x*_*im*_, *x*_*if*_) and the testing stage corresponds to *L*(*x*_*ij *_| *y*_*ij*_, *θ*', *x*_*im*_, *x*_*if*_).

The CLRT uses the linear mixed model *y*_*ij *_= *μ *+ *β*_0_*E*(*x*_*ij *_| *x*_*im*_, *x*_*if*_) + *z*_*i *_+ *ε *for the *L*(*y*_*ij *_| *θ *= (*μ*, *β*_0_, $\sigma_{e}^{2}$), *x*_*im*_, *x*_*if*_) part of the total likelihood. For the *L*(*x*_*ij *_| *y*_*ij*_, *θ*' = (*μ*', *β*'_0_, $\sigma {'}_{e}^{2}$), *x*_*im*_, *x*_*if*_) part of the likelihood, we used the model *logit*(Pr(*c*_*ij *_| *y*_*ij*_, *θ*')) = *μ*' + *β*'_0_*y*_*ij *_+ *ε*'_*ij *_[9]. Here, each child has one *c*_*ij *_value for each of its heterozygous parents with *c*_*ij *_= 1 if allele *A *was transmitted from that parent and *c*_*ij *_= 0 otherwise. This is not precisely analogous to the original two-step method testing stage where a simple linear model was used.

As in the two-stage method, the two parts of our likelihood are calculated under different models, so *β*_0 _and *β*'_0 _measure two different quantities. The null hypothesis in this method is that both *β*_0 _and *β*'_0 _are zero. A likelihood-ratio test statistic of the joint likelihood follows a $\chi_{2}^{2}$ distribution.

### Case study

As a case study, we analyzed Replicate 77 from the GAW15 Problem 3 data using CMMT, FBAT, CSST, CLRT, and Lange's two-stage method [4,5]. CMMT and FBAT analyses were performed using the PBAT version 3.2 software, and the CSST *p*-values were corrected for multiple-testing with a family-wise error rate (FWER) of *p *< 5.0 × 10^-8 ^and false discovery rate (FDR) of *p *< 0.05.

### Simulation analyses

To compute empirical type I error rates and power, we ran a similar analyses using CSST, CLRT, and the two-stage method on 100 partially-simulated and 82 simulated data sets from Problems 1 and 3.

## Results

### Case study results

Discrepancies were observed between the evidence of association from FBAT and CMMT analyses at the loci flanking the true susceptibility loci (Table 1). The one-stage CSST and CLRT analyses provided stronger evidence for association than either the CMMT or FBAT alone and the two-stage analysis.

**Table 1:** Case study *p*-values for SNPs flanking the causal loci

	*p*-Value (relative rank)			
	
SNP index	185	186	191	192
CMMT	4.0 × 10^-2 ^(457)	8.3 × 10^-5 ^(1)	1.2 × 10^-1 ^(1257)	2.6 × 10^-3 ^(43)
FBAT	9.9 × 10^-5 ^(2)	2.4 × 10^-3 ^(30)	4.4 × 10^-4 ^(5)	5.4 × 10^-3 ^(57)
2-stage^a^	1.0	2.4 × 10^-3 ^(1)	1.0	1.0
1-stage CSST	6.2 × 10^-5 ^(3)	4.3 × 10^-6 ^(1)	6.1 × 10^-4 ^(16)	2.2 × 10^-4 ^(7)
1-stage CLRT	8.0 × 10^-5 ^(3)	7.7 × 10^-6 ^(1)	6.9 × 10^-4 ^(13)	3.0 × 10^-4 ^(5)

^a^If a SNP is not among the top ten SNPs from the CMMT stage, it is not considered in the second stage, so the two-stage method *p*-value is listed as 1.0, the only *p*-value for which the null hypothesis will never be rejected, regardless of *α*.

The ranking of SNPs neighboring a causal locus varied considerably depending upon whether CMMT or FBAT scores are used. Performing a screening stage using only one of these measures is likely to miss causally linked SNPs. Specifically, in the two-stage design, SNPs 185, 191, and 192 are not considered for the second stage, so their high-ranking FBAT scores were not computed. In the CSST and CLRT one-stage methods, these SNPs did score relatively well, though they did not satisfy Bonferroni-corrected significance criteria.

### Problem 3 simulation results

The one-stage methods are empirically more powerful than the two-stage method based on comparisons in 82 Problem 3 simulated data sets (Table 2). The 813 SNPs on chromosome 2 were used to compute the type I error rate (giving 813 × 82 = 66,666 tests performed under the null hypothesis) while four SNPs adjacent to the causal loci on chromosome 9 were used to compute the empirical power. The type I error rates using each method were not statistically different from the nominal rate of 0.05/9187 = 5.4 × 10^-6^, although in testing whether our observed rate was different from the nominal rate, we did not take into account the dependence between tests that comes from linkage disequilibrium between chromosome 2 loci or from the fact that only 82 different sets of trait values were used. Table 2 shows the power at each adjacent SNP using the two-stage and single-stage methods. Power is greatest at a SNP in LD with simulated locus G (SNP 186). Though the other SNPs in Table 2 generally have low *p*-values, they are not low enough to pass the Bonferroni criterion.

**Table 2:** Problem 3 empirical power

	Power^a^			
	
SNP index	185	186	191	192
2-stage (*n *= 10)	0.01	0.66	0.01	0.00
1-stage CSST (*n *= 9187)	0.04	0.85	0.02	0.01
1-stage CLRT (*n *= 9187)	0.05	0.83	0.01	0.01

^a^Nominal type I error rate of *α *= 0.05, using a Bonferroni correction

### Problem 1 simulation results

Empirical power in this data set is consistently higher using the CLRT one-stage method (Tables 3 and 4) than when using the two-stage method. Even when exaggerating the multiple testing correction to *n *= 100,000, the CLRT one-stage method remains more powerful than the two-stage method (data not shown). The type I error rates for both methods are close to the rate expected given the *α *cutoff for significance and Bonferroni correction (data not shown).

**Table 3:** Problem 1 empirical power under the additive trait model with varying heritability and causal SNPs

	Power^a^			
	
	rs893613		rs1983011	
		
	*h*^2 ^= 0.25	*h*^2 ^= 0.5	*h*^2 ^= 0.25	*h*^2 ^= 0.5
2-stage (*n *= 10)	0.5	0.92	0.38	0.91
1-stage CLRT (*n *= 1509)	0.88	1	0.81	1

^a^Nominal type I error rate of *α *= 0.05, using a Bonferroni correction

**Table 4:** Problem 1 empirical power computed under the polygenic trait model power with varying heritability and proportion of genetic variance due to polygenes (*t*)

	Power^a^			
	
	t = 0.2		t = 0.5	
		
	*h*^2 ^= 0.5	*h*^2 ^= 0.8	*h*^2 ^= 0.5	*h*^2 ^= 0.8
2-stage (*n *= 10)	0.58	0.83	0.15	0.26
1-stage CLRT (*n *= 1509)	0.99	1	0.73	0.97

^a^Nominal type I error rate of *α *= 0.05, using a Bonferroni correction

## Discussion

Combining transmission and association information, as the two-stage method does, is more appealing than using either alone. However, the screening step in the current two-stage design selects SNPs based on top ranks without consideration of multiple testing and statistical significance. In these analyses we retained the top ten SNPs based on the recommendation of Van Steen et al. [5]. Retaining a larger number of SNPs in the screening stage would require a larger Bonferroni correction in the testing stage and could reduce power to detect a SNP's effect. For example, in the case study analyses, SNP 186 ranked first and was selected among the top ten in the screening step. In the testing step, SNP 186 was significant (FBAT *p*-value = 0.0024) after a Bonferroni correction of factor 10, however if 20 SNPs had been retained in the screening step, then SNP 186 would no longer be significant after a Bonferroni correction of factor 20. It is also important to note that causal loci may be missed if too few SNPs are selected in the screening stage. The two-stage method loses more power by not testing the vast majority of the SNPs than it gains in a smaller final multiple test correction. Our analysis demonstrates that these problems are addressed by using a single-stage test where both linkage and association contribute to final *p*-values with standard conservative Bonferroni correction.

The difference in power between the two methods is explained in part by the difference in the information each method uses. In the two-stage method, final conclusions are made using only the children's genotypes from families that are informative at the ten screenedSNPs. In contrast, the single-stage methods use all the children's genotypes. The single-stage methods use more information, allowing them more power. We note that applying the two-stage approach here to a data set with all children's genotypes available, provides no genotype cost savings. For population-level genome-wide association studies, two-stage approaches have been proposed as a means for reducing genotyping cost [10]. However, as the price of SNP genotyping falls and large publicly available SNP data sets gain popularity, it is increasingly important to find the most powerful method, regardless of genotyping costs.

The Problem 3 simulations allowed us to compare power while conditioning only on family structure. However, because the data were entirely simulated, some subtle aspects of LD may not be represented. Our comparisons based on Problem 1 data used real genotypes, so the LD structure is realistic; however the power we compute is conditional on these specific genotypes. Neither of these studies is ideal, but the fact that they have different strengths and weaknesses, yet produce very similar results supports our conclusions.

Both single-stage methods, CLRT and CSST, were found to havenominal type I error and comparable power. There remain several obvious improvements to our implementation of the CLRT single-stage method. In order to remain analogous to the original two-stage method, we used different genetic models in each part of the likelihood. A single genetic model including familial, polygenic, and environmental effects should be used for the entire likelihood.

It is important to note that all of these analyses were performed in the absence of population stratification. Transmission-based tests, like the FBAT, are robust to population structure. Since the final *p*-values from the two-step method are determined by the FBAT alone, the two-stage method is also robust to false positives caused by population structure. The CLRT and CSST one-stage methods, which combine transmission and association tests, are prone to false positives in the presence of population structure. In this case, both the CLRT and CSST approaches can be adjusted using a genomic control [11] or population structure [12] approach.

## Conclusion

We found that one-stage methods are empirically more powerful than the analogous two-stage method in two simulated data sets. Furthermore, when we investigated a particular simulation in detail, we found that causally linked SNPs often could be identified by either association or transmission, but not both. We conclude that rather than using only one of these methods to screen SNPs, the two should be used together, taking advantage of the strengths of each. In addition, it is sometimes believed that a large multiple testing problem will be reduced by using a two-stage screen and test method. Our results agree with previous results [6,13] that show that this is not the case. Thus, using a single-stage and standard multiple-test correction is more powerful than splitting the analysis into two stages.

## Competing interests

The author(s) declare that they have no competing interests.
